# Is withdrawing treatment really more problematic than withholding treatment?

**DOI:** 10.1136/medethics-2020-106330

**Published:** 2020-05-25

**Authors:** James Cameron, Julian Savulescu, Dominic Wilkinson

**Affiliations:** 1 Melbourne Law School, The University of Melbourne, Carlton, Victoria, Australia; 2 Murdoch Childrens Research Institute, Parkville, Victoria, Australia; 3 Oxford Uehiro Centre for Practical Ethics, University of Oxford, Oxford, UK; 4 Newborn Care, John Radcliffe Hospital, Oxford, UK

**Keywords:** allocation of healthcare resources, criminal law, clinical ethics, decision-making, distributive justice

## Abstract

There is a concern that as a result of COVID-19 there will be a shortage of ventilators for patients requiring respiratory support. This concern has resulted in significant debate about whether it is appropriate to withdraw ventilation from one patient in order to provide it to another patient who may benefit more. The current advice available to doctors appears to be inconsistent, with some suggesting withdrawal of treatment is more serious than withholding, while others suggest that this distinction should not be made. We argue that there is no ethically relevant difference between withdrawing and withholding treatment and that suggesting otherwise may have problematic consequences. If doctors are discouraged from withdrawing treatment, concern about a future shortage may make them reluctant to provide ventilation to patients who are unlikely to have a successful outcome. This may result in underutilisation of available resources. A national policy is urgently required to provide doctors with guidance about how patients should be prioritised to ensure the maximum benefit is derived from limited resources.

On 15 April 2020, the Medical Defence Union (MDU) in the UK provided guidance to members about the legal risks that they may face if they choose to remove a patient from a ventilator during the pandemic in order to provide ventilation to another patient who would obtain a greater benefit. The MDU advised ‘No action to withdraw life-saving treatment which is in the patient’s interests should occur unless the court first rules this is lawful’.^1^ This statement follows recent commentary^2^ and professional guidance^3^ that explicitly endorse the ethical permissibility of withdrawing life-sustaining treatment on the basis of resource allocation in the setting of overwhelming demand.

Under ordinary circumstances, withdrawing life-sustaining treatment that was in a patient’s interests may constitute homicide by omission. Referring such controversial decisions to a court would minimise the risk of subsequent legal or professional sanction. However, in this paper, we argue that differentiating between withdrawing and withholding treatment is ethically flawed. It is likely to result in inaction that would only interfere with the most effective use of limited resources. Decisions about the provision of ventilation in the situation envisaged will invariably need to be made quickly and when doctors are busy. If there is a choice between two options, the suggestion that an application must be made to the court before one course of action is taken will simply lead doctors to choose the other option. Discouraging withdrawal of treatment may also result in unnecessary pre-emptive rationing, as doctors may be concerned that they will not be able to withdraw treatment if they commence it. A national policy is required to guide the prioritisation of available resources.

## Potential situation

During the COVID-19 pandemic, it is possible that there will be a shortage of ventilators. If this situation arises, difficult decisions will need to be made about who should be treated with a ventilator and who should receive palliative care only. The issue has been extensively debated in relation to prioritising particular classes of patients over others. But these debates generally appear to assume the patients will be arriving at the hospital at the same time. The more likely scenario is that doctors would be required to juggle a range of patients needing ventilation at different times and for different durations. The following hypotheticals illustrate the difficulty doctors may face.

### Hypothetical 1 (John and Donald)

John is 75 and has a history of diabetes and chronic obstructive pulmonary disease with some decline in his overall health in recent years and several hospital admissions. He contracts COVID-19 and deteriorates quickly. John soon develops severe hypoxic respiratory failure. When he presented to hospital, it was uncertain whether John would survive this illness. However, he had capacity and expressed a strong wish to be provided with mechanical ventilatory support. At the time, there was sufficient availability of beds and ventilators in intensive care to accommodate John’s wishes, and he was intubated and admitted.

After 2 weeks of treatment in intensive care, John remains critically ill and dependent on high levels of support on the ventilator. The view of the intensive care team is that his overall chance of survival is low, <10%. If he is to survive, he will require a further prolonged period of treatment in intensive care.

During these 2 weeks, the COVID-19 virus has also continued to spread and hospitals are becoming concerned about a shortage of ventilators. Hospitals have started to increase the threshold for access to intensive care in anticipation of increased demand.

Donald is 73. He has several background health conditions, generally distrusts doctors and did not want to come to hospital. He struggles with symptoms of difficulty breathing for several days before calling an ambulance. When the ambulance officers arrive, he has a cardiac arrest and requires a prolonged period of resuscitation. He is stabilised by the time that he reaches the emergency department, but is assessed to have a chance of survival of <10%. Based on Donald’s chances of a successful outcome, the intensive care unit (ICU) doctors decide not to admit him to intensive care.

Donald is not provided ventilation because of his condition and prospect of recovery. Yet at the time this decision is made, John appears to have an equivalent chance of making a successful recovery. The only difference is that John is currently being ventilated.

### Hypothetical 2 (John and Arthur)

In the following days, the hospital experiences an intense surge of patients with respiratory failure. Despite expanding facilities (including using other critical care areas and operating theatres), the hospital has reached the point where there are no longer any available ventilators.

Arthur arrives at the hospital. Arthur is 55 and otherwise healthy, but has contracted COVID-19 and requires breathing support. Doctors estimate Arthur has a reasonably high chance of surviving with intensive care (>50%) and will require an intensive care stay of approximately 1 week (average for patients with COVID-19).^4^ They expect that he will return to full health quickly. But there are no available ventilators. Furthermore, all other hospitals in the region are also at capacity. Doctors feel that the only way to accommodate Arthur in intensive care is if they extubate John to free up a ventilator space.

## Potentially contradictory advice

In addressing concerns about limited resources and potential decisions to withdraw treatment, the MDU stated:

As the law currently stands, if a doctor is faced with the dilemma of competing interests between two patients and the possibility of withdrawing treatment which is in the patient’s best interests from that patient, in order to treat another patient, the doctor should first ensure their Trust makes an emergency application for a declaration to the Court of Protection.^1^

Following criticism that the Court of Protection hears applications in relation to the best interests of a patient, and not applications about the appropriate provision of resources, the advice was revised to suggest ‘an emergency Court application for a declaration’.^5^

The MDU statement appears to be at odds with the advice of the British Medical Association.^3^ The guidance produced by the British Medical Association recognises the difficult decisions that may need to be made and suggests ‘if there is radically reduced capacity to meet all serious health needs, it is both lawful and ethical for a doctor, following appropriate prioritisation policies, to refuse someone potentially life-saving treatment where someone else has a higher priority for the available treatment’.^3^ The British Medical Association suggests that there are no ethical differences between withdrawing and withholding treatment, but does not address whether there is a legal difference.

## Is a declaration required?

There is no general requirement to apply to the courts before withdrawing life-sustaining treatment from an adult.^6^ The MDU suggest that an application should be made for a declaration. It cannot be argued that it is necessary to obtain the remedy of a declaration before proceeding with an action. This is because a declaration allows the court to declare what the law is in relation to a situation; in making a declaration, the court does not alter what would be lawful. In relation to the criminal law, ‘the court has no power to authorise that which would otherwise be unlawful…. Nor can the court render unlawful that which would otherwise be lawful. The same is true in relation to a possible infringement of civil law’.^7^ The mere fact of obtaining a court declaration does not alter the lawfulness of the withdrawal of treatment. The withdrawal of treatment is either lawful or it is not and the court only has the power to identify this in a particular case. Through a declaration, the court does not ‘authorise’ treatment that would otherwise be unlawful.^8^

## Is it prudent to seek a court declaration?

While a declaration may not alter whether the proposed course of action is lawful, in what would be an unprecedented situation, it would be prudent to seek clarity from the court about what is appropriate. This declaration would not be sought to authorise a course of action but to identify whether it would be lawful. If ventilation is in the patient’s interests and their doctor withdraws that treatment, because of the doctor’s duty this omission could constitute murder.^9^ While the doctor clearly has no desire to cause the patient’s death, if they withdraw treatment with the knowledge this will inevitably result in death, legally the doctor may be taken to have intended the death.^10^ It must be recognised, however, that this omission would occur whether the doctor was withdrawing or withholding treatment.

In hypothetical 2 (John and Arthur), the doctor risks liability for homicide by omission regardless of whether they choose to withdraw or withhold treatment. In *Bland* Lord Browne-Wilkinson recognised in relation to criminal liability both removing a nasogastric tube and switching off a ventilator were positive acts but suggested ‘in neither case should the act be classified as positive, since to do so would be to introduce intolerably fine distinctions’.^11^ Instead, Lord Browne-Wilkinson held it should be recognised ‘what is being done is to omit to feed or to ventilate: the removal of the nasogastric tube or the switching off of a ventilator are merely incidents of that omission’.^11^ If withdrawing treatment is an omission to act, the situations of John and Arthur must be considered in the same way.

The situation appears to be analogous to the case of *Re A (Children) (conjoined twins: surgical separation*). The case involved conjoined twins. One twin, Jodie, would be capable of living a normal life if the twins were separated. The other twin, Mary, would die almost immediately as a result of the procedure. If the twins were not separated, both Jodie and Mary would die within 6 months. The Court of Appeal held the operation should be performed to separate the twins. It was recognised the operation was not in Mary’s best interests, but that it was the best available option. In relation to the potential criminal liability of the surgeon, Brooke LJ recognised that performing the surgery may constitute murder, but that the defence of necessity was available.^12^ Brooke LJ identified three conditions for necessity^13^:

- ‘the act is needed to avoid inevitable and irreparable evil’;
- ‘no more should be done than is reasonably necessary for the purpose to be achieved’;
- ‘the evil inflicted must not be disproportionate to the evil avoided’.

There is, however, at least one key difference between the situation under consideration and the case of Jodie and Mary. Jodie and Mary were inextricably linked, the position of each had to be understood in the context of the other. But in the situation being considered, it is somewhat artificial to suggest that the choice is between two people. In most situations, a hospital will have multiple ventilators and so there may be a whole range of potential scenarios. It may be argued that this makes the situation closer to *R v Dudley and Stephens*. The case involved three survivors of a shipwreck who had murdered and eaten a fourth crew member in order to live. It was held that an argument of necessity could not be made because the crew had made a conscious choice to kill the youngest and weakest crew member, ‘Was it more necessary to kill him than one of the grown men? The answer must be ‘No’’.^14^ Applied to the situation being considered, it may be argued that it is not necessary to differentiate between people in need of ventilation and there is no basis to measure the comparative value of lives.^15^ The difficulty with this argument is that it may actually be more necessary to deprive one person of ventilation than another. This is because one person’s condition may mean that they require a month of ventilation, whereas there may be four others who will only require a week.

The situation is even more complicated in relation to hypothetical 1 (John and Donald). There is a real possibility that treatment would be effective and it is difficult to argue that it is not in either John or Donald’s interests to pursue this opportunity. If there were still available ventilators but concern about future shortages, it would be difficult to argue that it was necessary to deny John and Donald treatment. This is because it could not be said that the death of other patients as a result of a shortage of ventilators was ‘inevitable’.

This discussion is not intended to resolve a potentially complex legal question. Instead, it is intended to demonstrate a range of issues arise and that the decision made and the basis for the decision may have serious legal consequences for a doctor. Given this, it may arguably be prudent to seek a declaration from the court in order to determine what would be lawful. If the unfortunate situation did arise in which there was a national shortage of ventilators, this does not mean that it would be necessary for applications to be made every time such a decision had to be made. Once the court had provided clear guidance about what would be lawful it would not be necessary to return to court to confirm this in every instance. It would also be completely impractical to suggest this should occur. For example, it would mean that if an ICU was full and there were no beds available elsewhere, each time there was a new patient requiring ventilation an application would need to be made to the court.

## Is differentiating ethically defensible?

It is ethically problematic to differentiate between a decision to withdraw treatment and a decision to withhold treatment, particularly when encouraging this distinction discourages the withdrawal of treatment. This risks causing deaths if the COVID-19 pandemic does result in a shortage of ventilators. Three key issues are discussed. First, it is indefensible to distinguish between withdrawing treatment from one patient and withholding treatment from another. Second, suggesting the withdrawal of ventilation is a more serious act than withholding treatment will discourage doctors from withdrawing treatment in cases in which this is appropriate. Third, if the distinction was acted on when there was a shortage of ventilators it would result in an increased number of deaths because decisions would be delayed and lives would be lost due to this inaction.

### Withdrawing versus withholding

It is easy to focus on the implications of withdrawing treatment from one patient, but the implications of not withdrawing ventilation from that patient must also be recognised. In a time of shortage, this may mean that treatment may need to be withheld from another patient. This is plainly problematic in relation to hypothetical 2, and also in relation to hypothetical 1 when the issue is properly framed.

In relation to hypothetical 2, the relevant question is which patient should receive life-sustaining treatment and which patient should be allowed to die. The fact that one patient arrived at the hospital first should not mean their life is given greater value or create a presumption in favour of maintaining their life. The principle of temporal neutrality states that the timing of an event cannot by itself be a morally relevant factor: when something occurs does not make a moral difference.^16^ Whether a patient presents to hospital earlier is not a morally relevant characteristic. Even strict equality would require that a coin be tossed to see who should receive treatment.

There is a commonly held intuition that the act of withdrawing treatment is different to withholding it because withdrawing treatment requires a positive act. But this position is difficult to justify. Whether a doctor withholds or withdraws treatment, they are making a decision not to provide treatment and this decision will deprive the patient of the opportunity to survive. Withdrawing and withholding treatment are morally equivalent.^17^

In relation to hypothetical 1, treatment is withheld from Donald on the basis of a criteria applied about the probability of success and the anticipated limited availability of ventilation. If the same criteria are applied to John, it suggests that ventilation should be withdrawn. The only difference is that John is currently receiving ventilation and Donald is not. Again, the fact that John arrived at the ICU earlier than Donald should not entitle him to preferential treatment. If criteria are applied for access to ICU, these criteria should be applied consistently for the sake of fairness.

Perhaps, the more difficult question in relation to hypothetical 1 is whether either Donald or John should be deprived of ventilation. Unlike in hypothetical 2, the patient is not denied treatment in order to immediately benefit another patient. Instead, treatment is not provided because of concern about the capacity to treat future hypothetical patients. This is not necessarily problematic, depending on the likelihood of this future shortage. If the future shortage was guaranteed, then there is little difference with the situation in hypothetical 2. But if, as is the current issue, the future is uncertain, it becomes more difficult to justify denying patients treatment in these circumstances. But this is more a question about the appropriate criteria for accessing ventilation and what level of pre-emptive rationing is appropriate. There is still no reason to differentiate between withdrawing and withholding treatment or between John and Donald.

### Discouraging doctors from withdrawing treatment

There is little difference between withdrawing and withholding treatment but the suggestion a court application is necessary before treatment may be withdrawn will create a strong bias in favour of withholding treatment from the new patient. From a practical perspective, doctors will avoid court because of the time and effort required to make a court application. But also the suggestion that one course of action requires court approval while the other does not suggests that one course is more extreme or serious than the other. It suggests that the conservative approach is to withhold treatment from the new patient. This may have a range of unjust consequences. For example, it may result in one person being ventilated for months while many others miss out on ventilation even when such ventilation is in their interests or before it becomes clear whether it is in their interests.

The other potential consequence of suggesting withdrawing treatment is a more serious course of action is that it will discourage a doctor from placing someone on a ventilator in the first place. This perverse incentive appears to arise in the situation described in hypothetical 1. In this situation, there are ventilators available, but uncertainty about the future availability of ventilators encourages doctors to ration these conservatively. One rationale for not taking Donald to intensive care (despite there currently being some beds available) is the concern that in the coming days ventilators will run out, but doctors will be unable to stop treatment for patients already in the ICU. A belief that a court application is required before ventilation may be withdrawn may leave doctors reluctant to ventilate people who are likely to require an extended period of ventilation in circumstances in which there is little chance of this being successful. Such an outcome is not in anyone’s interests. It means that ventilators will be underutilised and patients may be denied an opportunity to be ventilated because of concerns about shortages that may or may not eventuate.

Far more people will receive ventilation if an ICU is running at 100% capacity but ventilation is withdrawn from some patients than if an ICU is running at 80% capacity but ventilation is not withdrawn from patients once it is commenced. This would mean that in hypothetical 1, both John and Donald could be ventilated if there were available ventilators. But if the situation in hypothetical 2 then arose, ventilation could be withdrawn from one of them to accommodate Arthur. This gives everyone the greatest possible opportunity to receive ventilation. Differentiating between withdrawing and withholding treatment would inhibit such an approach because it discourages the withdrawal of treatment and so encourages pre-emptive rationing that may ultimately prove unnecessary.

### Delaying decisions causes deaths

If the MDU statement was followed, it is likely to result in more deaths and avoidable deaths. In the situation envisaged in hypothetical 2, the health system would be in crisis and choices like the choice between Arthur and John would need to be made every day and across the country. Even if the courts had the capacity to hear that many cases, the delay caused by preparing for and making a court application would result in patients deteriorating and dying while they waited for a decision about ventilation. This would not occur in the context of systematic decision-making to ensure the most effective use of resources. Instead, who received ventilation would be almost random, with those who arrived first having a greater opportunity.

Concern about this occurring is likely to further encourage hospitals to ration ventilators pre-emptively, in the manner described in the previous section. Such an approach will lead to underutilisation of available ventilators.

## How should decisions be made?

To ensure the most effective use of limited resources, a policy is required to clearly identify how patients should be prioritised. This will not only ensure the greatest possible benefit is derived from available treatments, but will also ensure rationing decisions are made by reference to the actual resources available. The Secretary of State or the Clinical Commissioning Groups may make policies that recognise limited resources and seek to maximise the effectiveness of the resources available to them.^18^ The *National Health Services (NHS) Act 2006* gives Secretary of State a wide discretion to determine what services will be provided and they are not limited to determinations based on clinical need.^19^ Such directions must be rational, proportionate and consistent with human rights.^20^ Rationing decisions occur every day in the NHS, including in circumstances that result in the death of patients.^21^

If doctors are left to make decisions about prioritisation on their own, they face great difficulty in justifying these decisions. This is because it would be up to the doctor to demonstrate that it was necessary to make a choice between patients. These decisions would be particularly difficult to justify in the scenario described in hypothetical 1. A national policy is urgently required to ensure that the maximum benefit may be derived from the limited number of ventilators that are available. This policy should not distinguish between withdrawing and withholding treatment in order to ensure ventilators are being used where ever possible and not lying idle in case of future increased demand.

## Data Availability

There are no data in this work.
